# Neurodevelopmental outcomes among 2- to 3-year-old children in Bangladesh with elevated blood lead and exposure to arsenic and manganese in drinking water

**DOI:** 10.1186/s12940-016-0127-y

**Published:** 2016-03-12

**Authors:** Ema G. Rodrigues, David C. Bellinger, Linda Valeri, Md Omar Sharif Ibne Hasan, Quazi Quamruzzaman, Mostofa Golam, Molly L. Kile, David C. Christiani, Robert O. Wright, Maitreyi Mazumdar

**Affiliations:** Department of Neurology, Boston Children’s Hospital, Boston, MA USA; Department of Environmental Health, Harvard T.H. Chan School of Public Health, Boston, MA USA; Department of Neurology, Harvard Medical School, Boston, MA USA; Department of Psychiatry (Biostatistics), McLean Hospital, Belmont, MA USA; Department of Psychiatry, Harvard Medical School, Boston, MA USA; Dhaka Community Hospital, Dhaka, Bangladesh; Department of Public Health, Oregon State University, Corvallis, OR USA; Department of Preventive Medicine, Mount Sinai School of Medicine, New York, NY USA

**Keywords:** Arsenic, Manganese, Lead, Cognitive function

## Abstract

**Background:**

The people of Bangladesh are currently exposed to high concentrations of arsenic and manganese in drinking water, as well as elevated lead in many regions. The objective of this study was to investigate associations between environmental exposure to these contaminants and neurodevelopmental outcomes among Bangladeshi children.

**Methods:**

We evaluated data from 524 children, members of an ongoing prospective birth cohort established to study the effects of prenatal and early childhood arsenic exposure in the Sirajdikhan and Pabna Districts of Bangladesh. Water was collected from the family’s primary drinking source during the first trimester of pregnancy and at ages 1, 12 and 20–40 months. At age 20–40 months, blood lead was measured and neurodevelopmental outcomes were assessed using a translated, culturally-adapted version of the Bayley Scales of Infant and Toddler Development, Third Edition (BSID-III).

**Results:**

Median blood lead concentrations were higher in Sirajdikhan than Pabna (7.6 vs. <LODμg/dL, *p* <0.0001) and water arsenic concentrations were lower (1.5 vs 25.7 μg/L, *p* <0.0001). Increased blood lead was associated with decreased cognitive scores in Sirajdikhan (β = −0.17, SE = 0.09, *p* = 0.05), whereas increased water arsenic was associated with decreased cognitive scores in Pabna (β = −0.06, SE = 0.03, *p* = 0.05). Water manganese was associated with fine motor scores in an inverse-U relationship in Pabna.

**Conclusion:**

Where blood lead levels are high, lead is associated with decreased cognitive scores on the BSID-III, and effects of other metals are not detected. In the setting of lower lead levels, the adverse effects of arsenic and manganese on neurodevelopment are observed.

**Electronic supplementary material:**

The online version of this article (doi:10.1186/s12940-016-0127-y) contains supplementary material, which is available to authorized users.

## Background

According to the most recent Bangladesh National Drinking Water Quality Survey of 2009, 13 % of 14,492 drinking water samples exceeded water arsenic (As) concentrations of 50 μg/L, the permissible limit in Bangladesh, exposing about 22 million people [1]. The adverse health effects associated with this epidemic include decreased lung function, skin lesions, and type 2 diabetes, among others [2–4]. Although fewer studies have addressed the adverse health effects of manganese (Mn), it is increasingly recognized that Mn exposure is also associated with adverse health effects and that high levels have been found in Bangladesh, with 42 % of tubewells tested exceeding the World Health Organization’s (WHO) former guideline of 400 μg/L (as of 2011, there is no WHO guideline for Mn) [5, 6]. However, a detailed survey of the distribution of As and Mn in the groundwater of Bangladesh, reported that high As and Mn concentrations are not necessarily correlated [6]. Exposure to lead (Pb) presents a third health threat prevalent in Bangladesh. While there are no published nationwide surveys on Pb exposures in Bangladesh, several studies have reported blood Pb levels >10 μg/dL, indicating high exposures among children [7–9].

While the neurotoxicity of Pb has been well documented in both children and adults [10], fewer studies have investigated the neurotoxic effects of As and Mn in children. It has been shown that concurrent drinking water As concentrations were negatively associated with cognitive function among children in Bangladesh [11, 12]. Others have found that urinary As was negatively associated with IQ and other cognitive measures in older children [13–16]. It has been shown that higher water Mn concentrations are associated with lower achievement scores, IQ, and behavioral scores among children [17–19]. Claus Henn et al. found a non-linear relationship between blood Mn concentrations at 12 months of age and 12-month mental development index with the inflexion point around 2.4 μg/dL [20] and others have found a similar non-linear relationship between hair and blood Mn and IQ [21].

Children are often exposed to multiple contaminants simultaneously, yet few studies have examined the interactions between As, Mn, and Pb. While some have found no association between exposure to multiple metals in children’s blood (i.e. lead, arsenic, cadmium, and manganese) and neurodevelopmental outcomes using latent class analysis [22], others have reported significant interactions between Mn and Pb in cord blood [23] and in children’s blood at 12 months of age [24] and 8- to 11 years of age [25], as well as interaction between As and Mn in children aged 11- to 13 years, using hair concentrations [26].

The objective of our current study was to investigate 1) the associations between environmental exposure to As, Mn, and Pb and neurodevelopmental outcomes at age 20 to 40 months in Bangladeshi children and 2) the potential interactions of As, Mn, and Pb on these outcomes.

## Methods

### Study design and participants

The women and children in this study were participants in a prospective birth cohort study conducted in the Sirajdikhan and Pabna regions of Bangladesh between 2008 and 2011, investigating the effects of arsenic-contaminated drinking water and reproductive health outcomes [27]. In that study, pregnant women were recruited from the Sirajikhan and Pabna Sadar Upazilas of Bangladesh. Gestational age was determined by first trimester (<16 weeks) ultrasound. When children were aged 12–40 months, we re-contacted the parents in the birth cohort study and invited them and their children to participate in this current study investigating the effects of prenatal and early childhood exposure to As, Mn, and Pb on early childhood development. This study included two visits, one at approximately 12 months of age and one at 20 to 40 months of age.

This study was approved by the Human Research Committees at the Harvard T.H. Chan School of Public Health and Dhaka Community Hospital, and informed consent was obtained from each mother prior to any data collection. The Institutional Review Board at Boston Children’s Hospital formally ceded review of this study to the Harvard Chan School.

### Questionnaires

At the 20 to 40-month follow-up visit, a questionnaire was administered to collect demographic information and maternal and child’s medical history. Additionally, a translated version of the Home Observation for Measurement of the Environment (HOME) instrument was administered at either visit to assess aspects of the emotional, social, and cognitive stimulation available to the children [28]. The Edinburgh Depression scale [29] and the Raven’s Matrices were also administered to mothers at the 20 to 40-month follow-up visit.

### Metal concentrations

Water samples from the tube well used by the family as the primary drinking water source were collected during the first trimester of pregnancy and follow-up visits at age 1 month, 12 months and 20 to 40 months. Approximately 50 ml of water was collected in a polyethylene tube and preserved with ultrapure nitric acid. Samples were shipped at room temperature to the Harvard Chan School where they were aliquoted and sent to Environmental Laboratory Services (North Syracuse, New York) or Spectrum Analytical Inc. (Agawam, MA) for analysis of As and Mn by inductively coupled plasma-mass spectrometry following US EPA method 200.8 [30]. Due to the high correlations among the concentrations at the different time points, the final regression analyses included only water As and Mn concentrations collected concurrently with the BSID-III assessment at 20–40 months.

Pb concentrations were measured in whole blood when the child was 20 to 40 months old using the LeadCare® II portable system (Magellan Diagnostics, Inc. North Billerica, MA, USA). The limit of detection for blood Pb was 3.3 μg/dL.

### Neurodevelopmental assessment

Neurodevelopmental outcomes were assessed at 20–40 months of age using a translated and culturally-adapted version of the Bayley Scales of Infant and Toddler Development, Third Edition (BSID-III). The BSID-III was administered at the Sirajdikhan and Pabna clinics by trained personnel. All sessions were videotaped and reviewed by a second administrator in Bangladesh. Approximately 5 % were reviewed by Dr. Bellinger as part of quality control. Age-adjusted Z-scores were calculated for the five domains (i.e. cognitive, receptive language, expressive language, fine motor and gross motor). Due to small numbers of children in certain age categories, we combined certain ages into one category for calculation of the Z-score. Specifically, we combined all children who were 19 to 22 months, 34 to 35 months, and 36 to 40 months of age.

### Statistical analyses

All statistical analyses were performed using SAS version 9.3 (SAS Institute, Inc., Cary, NC). Demographic characteristics were calculated for the entire cohort as well as for those who were included and excluded from the final regression model due to missing data. Chi-square tests were used to compare categorical variables, and Wilcoxon signed rank tests were used to compare continuous variables, including water and blood concentrations that were right-skewed. Due to the lognormal distributions of the water and blood measurements, metal concentrations were natural log transformed for use in the linear regression models. Linear regression was used to assess the relationship between water As and Mn and blood Pb concentrations and children’s age-adjusted BSID-III z-scores adjusting for several potential confounders identified by a review of the literature, including maternal and child characteristics such as maternal age, maternal education, exposure to environmental tobacco smoke, child’s sex, HOME score, maternal Raven score, and child’s hematocrit levels. Generalized additive models (GAM) were used to assess the shapes of the relationships between the exposure measures and the BSID-III z-scores to determine if additional terms (e.g. quadratic) would be appropriate in the regression models. Two-way interaction terms between the three exposures were also assessed to determine if the effect estimates of a single exposure differed by varying concentrations of an additional exposure.

## Results

The median age of the children at the time of neurodevelopmental testing was 2.3 years, with the ages ranging from 20 to 40 months. Of the 812 children who participated, 287 (35.3 %) children were excluded from analyses due to missing data. Specifically, 51 had no water data at 20 to 40 months, 181 had no fingerstick blood Pb concentration and 56 were missing data on covariates including hematocrit level (*n* = 42), maternal Raven score (*n* = 12), and HOME score (*n* = 1). More children from Sirajdikhan were excluded compared to Pabna (41.5 vs. 29.0 %, *p* = 0.0001) because the LeadCare II machine was temporarily not working at that site, and there were differences in maternal Raven score (*p* <0.0001), HOME score (*p* = 0.01), and gestational age (*p* = 0.0003) between the included and excluded groups (Table 1). Overall, children who were excluded from analyses had mothers who had less formal education, lower maternal Raven scores, and they had slightly higher HOME scores and gestational age, although the difference in gestational age was not clinically significant. When comparing maternal and child characteristics by clinic, there was no statistically significant difference in maternal age, maternal education, child’s age, or child’s sex, but there were statistically significant differences in maternal exposure to second-hand smoke, preterm birth, pica, and birth measurements (Additional file 1: Table S1). Specifically, there were more cesarean births, first births, and full-term births in Sirajdikhan compared to Pabna. Additionally, child weight was higher in Sirajdikhan whereas child length and head circumference were greater in Pabna. Mothers also reported more exposure to second-hand tobacco smoke in Pabna compared to Sirajdikhan.

**Table 1:** Demographics for study population and comparison of those excluded due to missing data

	Full study *N* = 812		Current analysis *n* = 525		Excluded *n* = 287		
	*n*	%	*n*	%	*n*	%	*p*-value
Clinic^a^							0.0002
Sirajdikhan	409	50.4	239	45.5	170	59.2	
Pabna	403	49.6	286	54.5	117	40.8	
Maternal characteristics							
Age at enrollment, median(range)^b^	22 (18–41)		22 (18–39)		22 (18–41)		0.69
Education^a^							0.06
Primary or less	380	46.8	233	44.4	147	51.2	
Secondary or greater	432	53.2	292	55.6	140	48.8	
Type of delivery^a^							0.10
Vaginal	517	63.7	347	66.1	170	59.2	
Cesarean	278	34.2	166	31.6	112	39.0	
Vaginal forceps assisted	17	2.1	12	2.3	5	1.8	
Exposed to second-hand smoke^a^							0.05
Yes	346	42.6	211	40.2	135	47.1	
No	465	57.3	314	59.8	151	52.6	
Missing	1	0.1	0	0.0	1	0.3	
Raven score, median (range)^b^	24 (0–54)		25 (3–54)		22 (0–46)		<0.0001
Missing	14		0		14		
Home score, median (range)^b^	43 (30–48)		43 (30–48)		44 (34–47)		0.01
Missing	2		0		2		
Child characteristics							
Child’s age at time of assessment in years, median (range)^b^	2.3 (1.7–3.4)		2.3 (1.7–3.3)		2.4 (1.9–3.4)		0.72
Sex^a^							0.43
Male	412	50.7	261	49.7	151	52.6	
Female	400	49.3	264	50.3	136	47.4	
Birth Order^a^							0.20
1	344	42.4	213	40.6	131	45.7	
2	282	34.7	189	36.0	93	32.4	
3	129	15.9	81	15.4	48	16.7	
4+	56	6.9	42	8.0	14	4.9	
Missing	1	0.1	0	0.0	1	0.3	
Preterm birth, <37 weeks^a^							0.28
Yes	176	21.7	120	22.9	56	19.5	
No	633	78.0	404	77.0	229	79.8	
Missing	3	0.4	1	0.2	2	0.7	
Pica^a^							0.75
Yes	314	38.7	205	39.0	109	38.0	
No	497	61.2	319	60.8	178	62.0	
Missing	1	0.1	1	0.2	0	0.0	
Gestational age in weeks, median (range)^b^	38.0 (28–42)		38.0 (28–42)		39.0 (29–42)		0.0003
Missing	3		1		2		
Birth Weight in kg, median (range)^b^	2.9 (0.8–4.8)		2.9 (0.8–4.5)		2.8 (1.0–4.8)		0.17
Current length in cm, median (range)^b^	83.0 (48–96)		82.0 (48–96)		83.0 (68–94)		0.46
Missing	1				1		
Current weight in kg, median (range)^b^	11.0 (6.1–20.0)		11.0 (6.1–20.0)		11.0 (7.3–17.8)		0.51
Missing	2				2		
Current head circumference in cm, median (range)^b^	45.0 (40–51)		45.0 (40–51)		45.0 (40–50)		0.16
Missing	1				1		
Current hematocrit, %, median (range)^b^	35 (18–58)		35 (18–56)		35 (22–58)		0.63
Missing	78		0		78		

^a^Chi-square test

^b^Wilcoxon rank sum test

There were statistically significant differences in As, Mn, and Pb exposures between the participants from the two clinics (Table 2). Overall, median water As concentrations were higher in Pabna compared to Sirajdikhan whereas median water Mn and blood Pb concentrations were higher in Sirajdikhan. The water As concentrations were particularly low in Sirajdikhan, where the majority (81 %) of water samples were below 50 μg/L, the drinking water standard in Bangladesh. In contrast, the majority (67 %) of blood Pb concentrations were below the limit of detection (LOD) in Pabna. Because of the many differences between exposure profile and distribution of confounders, the clinics were analyzed separately.

**Table 2:** Water and blood concentrations by clinic

	Sirajdikhan						Pabna						
Variable	*N*	% <LOD	P25	Median	P75	Maximum	*N*	%< LOD	P25	Median	P75	Maximum	*P*-value^a^
Water Arsenic, μg/L													
1st Trimester	238	29	LOD^b^	1.3	1.9	380	286	11	5.3	26.5	81	1400	<0.0001
1 month	238	49	LOD^b^	0.8	1.9	120	285	10	5.9	31	120	1400	<0.0001
12 months	92	40	LOD^b^	0.8	38.2	701	99	13	4.5	30.5	125	828	<0.0001
20–40 months	239	31	LOD^b^	1.5	21.6	653	286	9	4.4	25.7	130	1470	<0.0001
Water Manganese, μg/L													
1st Trimester	239	13	30	540	960	3300	286	1	270	500	900	3300	0.02
1 month	238	3	39	560	890	3900	278	0	340	545	1060	3900	0.0001
12 months	92	0	217	1150	2000	4650	99	1	244	464	891	2470	0.009
20–40 months	239	1	164	948	1820	4750	286	0	299	515	969	4050	0.003
Blood Lead, μg/dL	239	6	5.5	7.6	10.4	43	286	67	LOD^b^	LOD^b^	3.8	13.8	<0.0001

^a^Wilcoxon rank sum test

^b^LOD for water As = 0.5–0.75 μg/L_,_ LOD for water Mn = 0.5 μg/L, LOD for blood lead = 3.3 μg/dL

The water concentrations at the four different time points were highly correlated for both As (ρ = 0.77–0.88, *p* <0.0001) and Mn (ρ = 0.71–0.84, *p* <0.0001) in Pabna, although low correlations were observed in Sirajdikhan. The correlation between water As and Mn at 20 to 40 months was low in both Sirajdikhan (ρ = −0.06) and Pabna (ρ = 0.14). Additionally, the correlations between blood Pb concentrations and water As (ρ = 0.11 in Sirajdikhan, ρ = −0.10 in Pabna) and water Mn (ρ = 0.07 in Sirajdikhan, ρ = 0.05 in Pabna) were low, allowing us to include all exposures in the regression models simultaneously.

The age-adjusted BSID-III z-scores also differed by clinic with statistically significantly higher scores (*p* ≤0.01) in Sirajdikhan for all domains except receptive and expressive language, for which there was no statistically significant difference in scores between the two clinics. Due to the differences between clinics in exposure profiles, BSID-III scores, and covariates, all results are presented stratified by clinic.

**Fig. 1:**
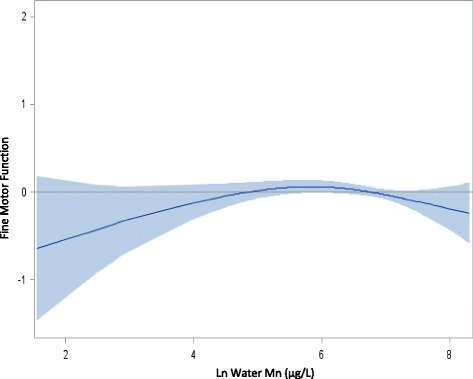
The effect of water Mn on BSID-III fine motor scores. Water Mn is associated with fine motor z-scores in an inverse-U relationship with an inflection point around 6 on Ln scale ≈ 400 μg/L

The results of the GAM indicated that most associations were linear between the exposures (As, Mn, and Pb) and the BSID-III z-scores, with the exception of water Mn concentrations and fine motor scores, for which an inverse-U shaped curve was observed (Fig. 1). With a statistically significant spline term (*p* = 0.03), we included a quadratic term for water Mn in the fine motor regression models in addition to the linear terms for water As and blood Pb. All other models included only linear terms for the exposures.

Results of the regression analyses are presented in Table 3. Results are presented only for cognitive and fine motor domains. No statistically significant associations were found between the three exposures and children’s scores on receptive and expressive language or gross motor domains. Our results show that blood lead concentrations were negatively associated with age-adjusted cognitive z-scores in Sirajdikhan (β = −0.17, SE = 0.09, *p* = 0.05), where blood lead concentrations were higher. In Pabna, where water As concentrations were higher, water As was inversely associated with age-adjusted cognitive z-scores (β = −0.06, SE = 0.03, *p* = 0.05). In contrast, no statistically significant association was observed with water Mn concentrations and cognitive scores in either Pabna or Sirajdikhan. While some associations between As, Pb, and cognitive scores mentioned above were statistically significant, the coefficients were not statistically different by clinic (As_Sirajdikhan vs. Pabna_ : *p* = 0.14, Mn _Sirajdikhan vs. Pabna_: *p* = 0.27, Pb _Sirajdikhan vs. Pabna_ : *p* = 0.25). Water Mn concentrations were associated with fine motor scores with an inverse-U relationship (β_Ln water Mn_^2^ = −0.08, SE = 0.03, *p* = 0.02) in Pabna. Our interpretation of the inverse-U relationship is that at low levels of manganese exposure (<6 on ln scale or approximately 400 μg/L), manganese is beneficial to fine motor development, whereas at higher levels manganese exposure is detrimental.

**Table 3:** Multivariate model between As, Mn, and Pb exposures and BSID-III scores at 20–40 months

	Cognitive				Fine motor			
	Sirajdikhan *n* = 239		Pabna *n* = 286		Sirajdikhan *n* = 239		Pabna *n* = 285	
Exposures	β (SE)	*p*-value	β (SE)	*p*-value	β (SE)	*p*-value	β (SE)	*p*-value
Ln Water As	−0.002 (0.02)	0.93	−0.06 (0.03)	**0.05**	−0.05 (0.03)	0.09	0.02 (0.03)	0.48
Ln Water Mn	0.02 (0.02)	0.35	−0.06 (0.07)	0.33	−0.04 (0.03)	0.21	0.85 (0.39)	0.03
Ln Water Mn2	-	-	-	-	-	-	−0.08 (0.03)	**0.02**
Ln Blood Pb	−0.17 (0.09)	**0.05**	0.02 (0.12)	0.87	0.07 (0.11)	0.50	−0.07 (0.11)	0.50

All models are adjusted for maternal age, maternal education, child’s gender, exposure to second-hand smoke, HOME score, maternal Raven score, and child hematocrit levels. Models use age-adjusted BSID-III scores

All exposures are included in the models simultaneously

*Ln* natural log, *SE* standard error, *As* arsenic, *Mn* manganese, *Pb* lead

To minimize the missing data on exposures and confounders in the multivariate models, we also imputed data for HOME scores and maternal Raven scores using multiple imputations, and we used the water concentrations measured at 1 month post-partum for those missing water concentrations at 20–40 months (Additional file 2: Table S2). Overall, the regression coefficients did not change. Due to the large number of missing blood Pb and hematocrit levels, we also conducted a sensitivity analysis by removing blood Pb and hematocrit from the models, leading to larger sample sizes (Additional file 2: Tables S3 and S4). While the regression coefficients did not change dramatically, the exclusion of blood Pb and hematocrit levels did reduce the power to observe a statistically significant association between As and cognitive scores in Pabna. Because Pb is such a significant predictor of cognitive function, we believe it is important to adjust for Pb in the models before interpreting the effects of As and Mn.

Of the interactions tested (i.e. As x Mn, As x Pb, and Mn x Pb), the only statistically significant interaction term was between As and Pb in relation to cognitive scores in Pabna (Table 4). The inverse slope of the association between water As concentration and cognitive scores was steeper as blood lead concentrations increased (β_AsxPb interaction_ = −0.15, *p* = 0.003), suggesting that the negative effect of As on cognitive function was exacerbated by increasing Pb concentrations. This finding should be considered with caution as most of the blood Pb concentrations in Pabna were below the LOD.

**Table 4:** Associations between As, Mn, and Pb exposures and Cognitive BSID-III at 20–40 months

	Cognitive			
	Sirajdikhan *n* = 239		Pabna *n* = 286	
	β (SE)	*p*-value	β (SE)	*p*-value
ln Water As	0.06 (0.08)	0.50	0.08 (0.05)	0.12
ln Water Mn	0.02 (0.02)	0.34	−0.05 (0.07)	0.42
ln Blood Pb	−0.16 (0.09)	0.07	0.45 (0.18)	0.01
Ln Water As*Ln Blood Pb	−0.03 (0.04)	0.47	−0.15 (0.05)	**0.003**

All models are adjusted for maternal age, maternal education, child’s gender, exposure to second-hand smoke, HOME score, maternal Raven score, and child hematocrit levels. BSID-scores are as age-adjusted z-scores

*Ln* natural log, *SE* standard error, *As* arsenic, *Mn* manganese, *Pb* lead

To investigate the effect of timing of exposure on children’s cognitive function, we conducted sensitivity analyses replacing the concurrent water As and Mn measurements with water measurements collected during the first trimester (Additional file 2: Table S5) and 1 month post-partum (Additional file 2: Table S6). Similar and slightly stronger associations were observed between first trimester water As concentrations and cognitive scores in Pabna (β = −0.09, SE = 0.03, *p* = 0.006), although no statistically significant association was observed between water As concentrations measrured 1 month post-partum and cognitive scores. It may be important to consider that most infants in this study were breastfed.

## Discussion

We observed associations between greater exposures to As, Mn, and Pb and decreased cognitive and motor function scores among children in two regions of Bangladesh with different overall exposure profiles. In Sirajdikhan, blood Pb concentrations were higher and water As concentrations tended to be lower compared to Pabna. In contrast, blood Pb concentrations were mostly below the LOD in Pabna and higher in Sirajdikhan. There was a wide distribution of water Mn in both regions.

In Sirajdikhan, where Pb levels were generally high, fingerstick blood Pb was associated with decreased cognitive scores. In contrast, the effect of Pb in Pabna, where levels were lower, was not statistically significant and relationships between other metals and neurodevelopment were found. In this setting, arsenic was associated with decreased cognitive scores and manganese exposure was associated with fine motor function in an inverse-U relationship, indicating increased motor function at concentrations <400 μg/L and decreased motor function at concentrations >400 μg/L.

Our findings are consistent with other studies that have reported negative associations between water As concentrations and reduced intellectual function among 6-year-old children exposed to high levels of As (mean = 120 μg/L) after adjustment for water Mn [11, 12], although we did not observe an association in Sirajdikhan, where water As concentrations were significantly lower. We also did not observe an association between water As and language scores or motor scores, although others have reported negative associations between urinary As and verbal abilities and long-term memory [31] as well as lower motor function scores associated with higher As exposures [32].

While we did not find an association between water Mn and cognitive scores, we did observe an association with fine motor scores, which is consistent with research conducted among welders exposed to Mn fumes [33, 34]. A decrease in motor function associated with Mn intake from water has also been observed among children [35]. Unlike the linear relationship reported by Oulhote et al., however, the relationship between water Mn and motor function in this study was an inverse-U relationship, indicating a beneficial effect of increased Mn at lower concentrations and the negative effects at higher concentrations similar to other studies [24, 36]. This finding is consistent with Mn being a beneficial nutrient as well as a neurotoxicant. It may be that such a finding would be more pronounced in undernourished populations, such as Bangladesh. Given that Mn homeostasis is tightly regulated through import and export proteins in humans, it is possible that a biomarker such as blood or hair may provide a better measure of physiologically active Mn than water [37].

Unlike previous studies that used blood or hair measurements for Mn, we did not find an interaction between Mn and Pb [23, 24] or Mn and As [26], but we did observe an interaction between As and Pb on cognitive scores in Pabna, where water As concentrations were higher and Pb concentrations were low. With the availability of measurements on multiple neurotoxicants, we were able to assess the effect of co-exposures and potential effect modification of individual exposures, but our sample sizes were quite small in the two regions, making it difficult to observe statistically significant multiple interactions in this study. A major strength of this study was the ability to study children in two different regions of Bangladesh with different exposure and demographic profiles. We had the unique opportunity to investigate the effects of combinations of both low and high environmental exposures on neurodevelopmental outcomes. We also had water As and Mn measurements at several time points, including the first trimester of pregnancy and 1 month post-partum, although our results focus on water measures concurrent with the BSID-III’s assessment and blood lead concentrations.

A limitation of this current study is the lack of a biomarker of exposure for As and Mn. While water As and Mn concentrations were available, we were unable to calculate intake for the children, and we did not consider other sources of exposure such as food. This potential for exposure misclassification is likely to be non-differential and bias our results towards the null. It is possible that a biomarker of cumulative exposure such as blood, hair or toenails may be more appropriate. One study conducted among older children (median age = 14 years) in Cambodia found that hair As concentrations were negatively associated with several neurobehavioral outcomes after adjusting for hair Mn, Pb, and cadmium (Cd) concentrations [38]. Additionally, while we had water As and Mn concentrations at several time points prior to the time of the BSID-III’s assessment, the high collinearity of repeated measurements of a chemical, particularly in Pabna, made it difficult to include all the measurements in our regression models. Our future work will include the analysis of children’s toenails as a biomarker of exposure to As and Mn, minimizing exposure misclassification, and will potentially include Bayesian statistical methods to account for correlated repeated exposure measures. Additional work will also consider the impact of children’s growth and nutritional status on these observed associations. When investigating the effect of environmental contaminants on health, it is important to investigate the effect of multiple exposures that may exist simultaneously as the effects may differ in the presence or absence of various contaminants.

## Conclusions

Where blood Pb levels are high, Pb is associated with decreased cognitive scores on the BSID-III, and effects of other metals are not detected. In the setting of lower Pb levels, the adverse effects of As and Mn on neurodevelopment are observed. Specifically, As is primarily associated with reduced intellectual functioning while Mn is primarily associated with decreased fine motor function. Further study of biomarkers of exposure, such as hair and nails, may expand these findings.

## Additional files

Additional file 1: Table S1. Comparison of covariates by clinic. (DOCX 21 kb) Additional file 2: Table S2. Multivariate model between As, Mn, and Pb and BSID-III scores at 20–40 months. **Table S3.** Multivariate model between As, Mn, and Pb and BSID-III scores at 20–40 months. **Table S4.** Multivariate model between As, Mn, and Pb and Bayley z-scores at 20–40 months. **Table S5.** Multivariate model between As and Mn (early pregnancy) and Pb exposures and Bayley z-scores at 20–40 months. **Table S6.** Multivariate model between As, Mn (1 month post-partum) and Pb and Bayley z-scores at 20–40 months. (DOCX 25 kb)

## Abbreviations

As: arsenic
Mn: manganese
WHO: World Health Organization
Pb: lead
IQ: intelligence quotient
HOME: home observation for measurement of the environment
EPA: environmental protection agency
BSID-III: Bayley Scales of Infant and Toddler Development, Third Edition
GAM: Generalized additive models
LOD: limit of detection
SE: standard error
