# Supplementary material for: Neurodevelopmental outcomes among 2- to 3-year-old children in Bangladesh with elevated blood lead and exposure to arsenic and manganese in drinking water
Source: Environ Health. 2016 Mar 12;15:44. doi: 10.1186/s12940-016-0127-y (PMC4788832; doi:10.1186/s12940-016-0127-y)
Supplement: Additional file 1: Table S1. — Comparison of covariates by clinic. (DOCX 21 kb) [file 12940_2016_127_MOESM1_ESM.docx]

| **Additional file 1: Table S1: Comparison of covariates by clinic** | | | | | |
| --- | --- | --- | --- | --- | --- |
|  | **Sirajdikhan**  **n=238** | | **Pabna**  **n=286** | | **Site Comparison** |
|  | **n** | **%** | **n** | **%** | **p-value** |
| **Maternal Characteristics** |  |  |  |  |  |
| Age at enrollment, median(range)^b^ | 22 (18-39) | | 22 (18-38) | | 0.78 |
|  |  |  |  |  |  |
| Education^a^ |  |  |  |  | 0.52 |
| Primary or less | 109 | 45.8 | 123 | 43.0 |  |
| Secondary or greater | 129 | 54.2 | 163 | 57.0 |  |
|  |  |  |  |  |  |
| Type of Delivery^a^ |  |  |  |  | <0.0001 |
| Vaginal | 102 | 42.9 | 244 | 85.3 |  |
| Cesarean | 134 | 56.3 | 32 | 11.2 |  |
| Vaginal Forceps Assisted | 2 | 0.8 | 10 | 3.5 |  |
|  |  |  |  |  |  |
| Exposed to Second-hand Smoke^a^ |  |  |  |  | <0.0001 |
| Yes | 74 | 31.1 | 137 | 47.9 |  |
| No | 164 | 68.9 | 149 | 52.1 |  |
|  |  |  |  |  |  |
| Raven Score, median (range)^b^ | 20 (6-54) | | 26.5 (3-39) | | <0.0001 |
|  |  |  |  |  |  |
| HOME Score, median (range)^b^ | 44 (38-48) | | 41.5 (30-47) | | <0.0001 |
|  |  |  |  |  |  |
| **Child Characteristics** |  |  |  |  |  |
| Child's Age at Time of Assessment in Years, median (range)^b^ | 2.3 (2.0-3.0) | | 2.3 (1.7-3.3) | | 0.01 |
|  |  |  |  |  |  |
| Sex^a^ |  |  |  |  | 0.67 |
| Male | 121 | 50.8 | 140 | 49.0 |  |
| Female | 117 | 49.2 | 146 | 51.0 |  |
|  |  |  |  |  |  |
| Birth Order^a^ |  |  |  |  | 0.03 |
| 1 | 111 | 46.6 | 101 | 35.3 |  |
| 2 | 79 | 33.2 | 110 | 38.5 |  |
| 3 | 35 | 14.7 | 46 | 16.1 |  |
| 4+ | 13 | 5.5 | 29 | 10.1 |  |
|  |  |  |  |  |  |
| Preterm Birth^a^ |  |  |  |  | <0.0001 |
| Yes | 13 | 5.5 | 107 | 37.4 |  |
| No | 225 | 94.5 | 178 | 62.2 |  |
| Missing | 0 | 0.0 | 1 | 0.3 |  |
|  |  |  |  |  |  |
| Pica^a^ |  |  |  |  | <0.0001 |
| Yes | 137 | 57.6 | 68 | 23.8 |  |
| No | 101 | 42.4 | 218 | 76.2 |  |
|  |  |  |  |  |  |
| Gestational Age in Weeks, median (range)^b^ | 39 (28-42) | | 37 (28-40) | | <0.0001 |
|  |  |  |  |  |  |
| Birth Weight in kg, median (range)^b^ | 2.9 (1.8-3.5) | | 2.8 (0.8-4.5) | | <0.0001 |
|  |  |  |  |  |  |
| Current Length at 24 Months in cm, median (range)^b^ | 82 (48-96) | | 83 (69-92) | | <0.0001 |
|  |  |  |  |  |  |
| Current Weight at 24 Months in kg, median (range)^b^ | 11 (6.1-20.0) | | 10.6 (7.8-15.0) | | 0.0009 |
|  |  |  |  |  |  |
| Current Head Circumference at 24 Months in cm, median (range)^b^ | 45 (42-49) | | 46 (40-51) | | <0.0001 |
|  |  |  |  |  |  |
| Current Hematocrit, %, median (range)^b^ | 36 (38-50) | | 34 (36-56) | | <0.0001 |
| ^a^Chi-square test |  |  |  |  |  |
| ^b^Wilcoxon rank sum test |  |  |  |  |  |
