# Supplementary material for: Neurodevelopmental outcomes among 2- to 3-year-old children in Bangladesh with elevated blood lead and exposure to arsenic and manganese in drinking water
Source: Environ Health. 2016 Mar 12;15:44. doi: 10.1186/s12940-016-0127-y (PMC4788832; doi:10.1186/s12940-016-0127-y)
Supplement: Additional file 2: Table S2. — Multivariate model between As, Mn, and Pb and BSID-III scores at 20–40 months. Table S3. Multivariate model between As, Mn, and Pb and BSID-III scores at 20–40 months. Table S4. Multivariate model between As, Mn, and Pb and Bayley z-scores at 20–40 months. Table S5. Multivariate model between As and Mn (early pregnancy) and Pb exposures and Bayley z-scores at 20–40 months. Table S6. Multivariate model between As, Mn (1 month post-partum) and Pb and Bayley z-scores at 20–40 months. (DOCX 25 kb) [file 12940_2016_127_MOESM2_ESM.docx]

**Additional file 2**

| **Table S2:Multivariate model between As, Mn, and Pb and BSID-III scores at 20-40 months** | | | | | | | |  |
| --- | --- | --- | --- | --- | --- | --- | --- | --- |
|  | **Cognitive** | | | | **Fine Motor** | | | |
|  | **Sirajdikhan n=265** | | **Pabna n=316** | | **Sirajdikhan n=265** | | **Pabna n=316** | |
| **Exposures** | **β (SE)** | **p-value** | **β (SE)** | **p-value** | **β (SE)** | **p-value** | **β (SE)** | **p-value** |
| Ln Water As | 0.004 (0.02) | 0.85 | -0.05 (0.03) | **0.08** | -0.04 (0.03) | 0.1 | 0.02 (0.03) | 0.56 |
| Ln Water Mn | 0.03 (0.02) | 0.23 | -0.09 (0.07) | 0.18 | -0.04 (0.03) | 0.19 | 0.80 (0.38) | 0.03 |
| Ln Water Mn2 | - | - | - | - | - | - | -0.07 (0.03) | **0.02** |
| Ln Blood Pb | -0.20 (0.08) | **0.02** | 0.05 (0.12) | 0.64 | 0.06 (0.10) | 0.54 | -0.07 (0.11) | 0.54 |
| Note: All models are adjusted for maternal age, maternal education, child's gender, exposure to second-hand smoke, HOME score, maternal Raven score, and child hematocrit levels. Models use age-adjusted BSID-III scores. | | | | | | | | |
| Note: All exposures are included in the models simultaneously and missing values were imputed for HOME scores, maternal Raven scores, and water concentrations. | | | | | | | | |

| **Table S3:Multivariate model between As, Mn, and Pb and BSID-III scores at 20-40 months** | | | | | | | | | | |  | |
| --- | --- | --- | --- | --- | --- | --- | --- | --- | --- | --- | --- | --- |
|  | **Cognitive** | | | | | **Fine Motor** | | | | | | |
|  | **Sirajdikhan n=380** | | **Pabna n=365** | | | **Sirajdikhan n=380** | | | | **Pabna n=364** | | |
| **Exposures** | **β (SE)** | **p-value** | **β (SE)** | **p-value** | | **β (SE)** | | **p-value** | | **β (SE)** | | **p-value** |
| Ln Water As | -0.02 (0.02) | 0.30 | -0.03 (0.03) | 0.28 | | -0.03 (0.02) | | 0.11 | | 0.04 (0.02) | | 0.14 |
| Ln Water Mn | 0.02 (0.02) | 0.30 | -0.08 (0.05) | 0.11 | | -0.01 (0.02) | | 0.77 | | -0.15 (0.05) | | **0.002** |
| Note: All models are adjusted for maternal age, maternal education, child's gender, exposure to second-hand smoke, HOME score, and maternal Raven score. Models use age-adjusted BSID-III scores. | | | | | | | | | | | | |
| Note: All exposures are included in the models simultaneously | | | | |  | |  | |  | |  | |

|  | |  |  | |  | |  | |  |  |  |
| --- | --- | --- | --- | --- | --- | --- | --- | --- | --- | --- | --- |
| **Table S4:Multivariate model between As, Mn, and Pb and Bayley z-scores at 20-40 months** | | | | | | | | | | |  |
|  | **Cognitive** | | | | | | | **Fine Motor** | | | |
|  | **Sirajdikhan n=409** | | | **Pabna n=403** | | | | **Sirajdikhan n=409** | | **Pabna n=402** | |
| **Exposures** | **β (SE)** | **p-value** | | **β (SE)** | | **p-value** | | **β (SE)** | **p-value** | **β (SE)** | **p-value** |
| Ln Water As | -0.02 (0.02) | 0.43 | | -0.03 (0.03) | | 0.32 | | -0.03 (0.02) | 0.10 | 0.03 (0.02) | 0.24 |
| Ln Water Mn | 0.02 (0.02) | 0.25 | | -0.08 (0.05) | | 0.14 | | -0.01 (0.02) | 0.65 | -0.15 (0.05) | **0.002** |
| Note: All models are adjusted for maternal age, maternal education, child's gender, exposure to second-hand smoke, HOME score, and maternal Raven score. Models use age-adjusted BSID-III scores. | | | | | | | | | | | |
| Note: All exposures are included in the models simultaneously, and missing values were imputed for HOME scores, maternal Raven scores, and water concentrations. | | | | | | | | | | | |

| **Table S5:Multivariate model between As and Mn (early pregnancy) and Pb exposures and Bayley z-scores at 20-40 months** | | | | | | | | | | | | | | | | |  |
| --- | --- | --- | --- | --- | --- | --- | --- | --- | --- | --- | --- | --- | --- | --- | --- | --- | --- |
|  | | **Cognitive** | | | | | | | | **Fine Motor** | | | | | | | |
|  | | **Sirajdikhan n=261** | | | | **Pabna n=304** | | | | **Sirajdikhan n=261** | | | | **Pabna n=303** | | | |
| **Exposures** | | **β (SE)** | | **p-value** | | **β (SE)** | | **p-value** | | **β (SE)** | | **p-value** | | **β (SE)** | | **p-value** | |
| Ln Water As 1^st^ trimester | | 0.009 (0.03) | | 0.78 | | -0.09 (0.03) | | **0.006** | | 0.07 (0.04) | | 0.10 | | 0.007 (0.03) | | 0.81 | |
| Ln Water Mn 1^st^ trimester | | 0.008 (0.02) | | 0.67 | | -0.09 (0.06) | | 0.13 | | 0.004 (0.02) | | 0.85 | | -0.10 (0.05) | | 0.08 | |
| Ln Water Mn2 | | - | | - | | - | | - | | - | | - | | - | | **-** | |
| Ln Blood Pb | | -0.18 (0.08) | | **0.03** | | 0.03 (0.12) | | 0.8 | | 0.02 (0.10) | | 0.85 | | -0.11 (0.11) | | 0.32 | |
| Note: All models are adjusted for maternal age, maternal education, child's gender, exposure to second-hand smoke, HOME score, maternal Raven score, and child hematocrit levels. Models use age-adjusted BSID-III scores. | | | | | | | | | | | | | | | | |  |
| Note: All exposures are included in the models simultaneously | | | | | | | | |  | |  | |  | |  | |  |
|  |  | |  | |  | |  | |  | |  | |  | |  | |  |

| **Table S6:Multivariate model between As, Mn (1 month post-partum) and Pb and Bayley z-scores at 20-40 months** | | | | | | | |  |
| --- | --- | --- | --- | --- | --- | --- | --- | --- |
|  | **Cognitive** | | | | **Fine Motor** | | | |
|  | **Sirajdikhan n=261** | | **Pabna n=296** | | **Sirajdikhan n=261** | | **Pabna n=295** | |
| **Exposures** | **β (SE)** | **p-value** | **β (SE)** | **p-value** | **β (SE)** | **p-value** | **β (SE)** | **p-value** |
| Ln Water As 1 month post-partum | 0.01 (0.04) | 0.72 | -0.05 (0.03) | 0.17 | 0.02 (0.05) | 0.69 | 0.03 (0.03) | 0.38 |
| Ln Water Mn 1 month post-partum | -0.06 (0.03) | **0.02** | -0.11 (0.07) | 0.11 | -0.03 (0.03) | 0.29 | -0.05 (0.07) | 0.41 |
| Ln Water Mn2 | - | - | - | - | - | - | - | - |
| Ln Blood Pb | -0.17 (0.08) | **0.04** | 0.05 (0.12) | 0.70 | 0.03 (0.10) | 0.77 | -0.09 (0.11) | 0.42 |
| Note: All models are adjusted for maternal age, maternal education, child's gender, exposure to second-hand smoke, HOME score, maternal Raven score, and child hematocrit levels. Models use age-adjusted BSID-III scores. | | | | | | | | |
| Note: All exposures are included in the models simultaneously | | | | |  |  |  |  |
